# Association of immune-mediated inflammatory diseases with depression and anxiety in patients with type 2 diabetes: A nationwide population-based study

**DOI:** 10.3389/fmed.2023.1103911

**Published:** 2023-04-17

**Authors:** Oh Chan Kwon, Yuna Kim, Jaeyoung Chun, Kyungdo Han, Min-Chan Park, Ryul Kim, Jie-Hyun Kim, Young Hoon Youn, Hyojin Park

**Affiliations:** ^1^Division of Rheumatology, Department of Internal Medicine, Yonsei University College of Medicine, Seoul, Republic of Korea; ^2^Division of Gastroenterology, Department of Internal Medicine, Yonsei University College of Medicine, Seoul, Republic of Korea; ^3^Department of Statistics and Actuarial Science, Soongsil University, Seoul, Republic of Korea; ^4^Department of Neurology, Inha University Hospital, Incheon, Republic of Korea

**Keywords:** immune-mediated inflammatory diseases, type 2 diabetes, depression, anxiety, risk

## Abstract

**Objective:**

Patients with type 2 diabetes (T2DM) are at a high risk of developing depression and anxiety. To better stratify the risk, we aimed to assess whether the presence of immune-mediated inflammatory diseases (IMIDs) confers a higher risk of depression and anxiety in these patients.

**Methods:**

Patients with T2DM without prior depression or anxiety who underwent national health examination between 2009 and 2012 (*n* = 1,612,705) were enrolled from the nationwide health check-up data from Korean National Health Insurance Service. The outcome events were incident depression and anxiety, defined as International Classification of Diseases, 10th Revision codes F32–F33 and F40–F41, respectively. Multivariable Cox proportional hazard regression analyses were conducted to estimate the adjusted hazard ratio (aHR) and 95% confidence interval (CI) according to the existence of IMIDs.

**Results:**

Over an average follow-up time of 6.4 years, existence of gut IMIDs was associated with a higher risk of depression (aHR: 1.28 [95% CI: 1.08–1.53]) and anxiety (1.22 [1.06–1.42]). Existence of joint IMIDs was associated with a higher risk of depression (1.34 [1.31–1.37]) and anxiety (1.31 [1.29–1.34]). Existence of skin IMID was associated with a higher risk of depression (1.18 [1.14–1.23]) and anxiety (1.13 [1.09–1.16]). The effect sizes of IMIDs on depression and anxiety were larger in those with ≥ 2 IMIDs (1.42 [1.19–1.69] and 1.49 [1.29–1.72], respectively) than in those with one IMID (1.30 [1.27–1.32] and 1.26 [1.24–1.28], respectively).

**Conclusion:**

In patients with T2DM, presence of IMIDs was associated with a higher risk of depression and anxiety. More stringent attention and screening for anxiety and depression should be encouraged in patients with T2DM and comorbid IMIDs due to clinical implications of psychological distress on patient-reported outcomes and prognosis.

## Introduction

Patients with type 2 diabetes (T2DM) are at higher risk of having depression and anxiety, compared with those without T2DM, as reflected by odds ratios of 1.67 and 1.73, respectively (1, 2). Depression and anxiety are important factors that adversely affect treatment outcomes and quality of life in patients with T2DM (3). Therefore, analyzing the risk of depression and anxiety in patients with T2DM, and closer screening for depression and anxiety in high risk patients are crucial.

Immune-mediated inflammatory diseases (IMIDs) are a group of heterogeneous diseases causing chronic inflammation and organ damage (4). IMIDs can be classified into three groups based on the affected organs. Inflammatory bowel diseases (IBD: Crohn’s disease and ulcerative colitis), inflammatory arthritides (rheumatoid arthritis and ankylosing spondylitis), and psoriasis are IMIDs that mainly affect the gut, joints, and skin, respectively (5–15). Studies have shown that each IMID is associated with a higher risk of depression and anxiety in the general population with effect sizes similar to that of T2DM (16–19). A population-based study reported a 2-fold higher risk of depression and a 1.6-fold higher risk of anxiety in patients with IBD (16). With regard to IMIDs that predominantly affect the joints, patients with rheumatoid arthritis had a 1.5-fold and 1.2-fold higher risk of depression and anxiety, respectively, compared to the controls (17), and patients with ankylosing spondylitis had a 1.7-fold and 1.8-fold higher risk of depression and anxiety, respectively (18). A higher risk of depression and anxiety has also been reported in patients with psoriasis compared with controls (1.4-fold and 1.3-fold higher risk of depression and anxiety, respectively) (19).

However, as the previous studies showing association between IMIDs and risk of depression and anxiety used general population as a control (16–19), it is unclear if the existence of IMIDs also confer an additional risk of depression and anxiety when limited to the patients with T2DM. Considering that low grade inflammation is present in patients with T2DM (20), it is of particular interest to analyze the possible role of IMIDs for depression and anxiety in T2DM. It is important to elucidate the effect of the existence of IMIDs on the risk of depression and anxiety in patients with T2DM, which can lead to a better stratification of the risk in these patients. Herein, we conducted a nationwide, population-based study to assess the association between the presence of IMIDs and the risk of depression and anxiety in patients with T2DM.

## Patients and methods

### Data source

We used nationwide, population-based data from the Korean National Health Insurance Service (NHIS) claims database. Comprehensive data including the socioeconomic status, demographics, medical procedures and treatments, diagnosis according to the International Classification of Diseases, 10th Revision (ICD-10), and rare intractable disease (RID) registration information (21) are provided by the NHIS. In the RID registration system, a diagnosis is thoroughly reviewed by the NHI and the corresponding healthcare institution. The data source have been described in detail previously (22). All individuals registered in the NHIS database are recommended to get their national health check-ups every 2 years. The health examination data include: blood pressure (BP); anthropometric data; and laboratory data, such as fasting serum glucose levels, creatinine levels, and cholesterol levels. Data on previous medical history and lifestyle factors, including smoking status, alcohol consumption, and physical activity, all of which were based on standardized self-reporting questionnaires, were also available.

### Study cohort

**Figure 1:**
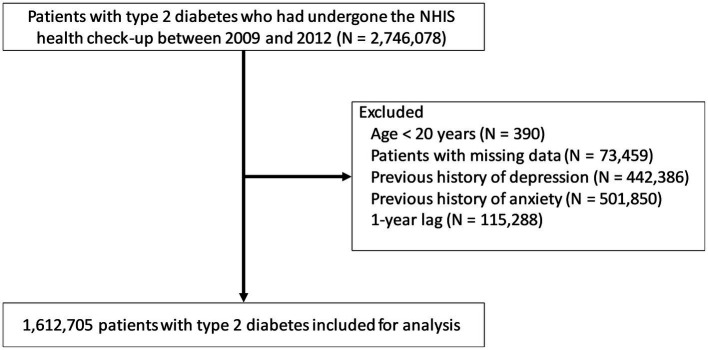
Selection of the study population. NHIS, National Health Insurance Service.

Initially, 2,746,078 patients with T2DM who had underwent the national health check-up between January 2009 and December 2012 were selected. Patients with T2DM were defined as those with ICD-10 codes of E11–14 with prescriptions for anti-diabetic medications or as those with a fasting plasma glucose ≥ 126 mg/dl (23, 24). The following subjects were excluded (Figure 1): (a) age < 20 years (*n* = 390); (b) missing data (*n* = 73,459); (c) previous history of depression (individuals with ICD-10 codes F32–F33 prior to the index date; *n* = 442,386); (d) previous history of anxiety (individuals with ICD-10 codes F40–F41 prior to the index date; *n* = 501,850); and (e) depression or anxiety or death that occurred within a year from the first health check-up date between January 2009 and December 2012 (*n* = 115,288). The remaining 1,612,705 patients with T2DM were included in the analysis. They were followed up from the baseline (the first health check-up date between January 2009 and December 2012) to the date of incident depression or anxiety, or December 2018, whichever came first.

### Definition of covariates and outcomes

Covariates were defined as previously reported: Crohn’s disease, ICD-10 code K50 + RID code V130 (24–27); ulcerative colitis, ICD-10 code K51 + RID code V131 (24–27); rheumatoid arthritis, ICD-10 code M05 or M06 + prescriptions for disease-modifying anti-rheumatic drugs (24, 28, 29); ankylosing spondylitis, ICD-10 code M45 + RID code V140 (24, 30); and psoriasis, ICD-10 code L40 (24, 31). A multicenter validation study of diagnostic algorithms for Crohn’s disease and ulcerative colitis showed that sensitivity, specificity, positive predictive and negative predictive values for the identification of patients with IBD were 92.1, 97.2, 96.4, and 93.7%, respectively (32). The definition of rheumatoid arthritis identified patients with definite rheumatoid arthritis with 96.5% for sensitivity, 92.4% for positive predictive value, and 58.7% for specificity (33). Diagnostic sensitivity, specificity, and positive predictive value of the definition for psoriasis were 96.2, 91.0, and 78.0%, respectively (34). As there was no nationwide validation study conducted for the definition of ankylosing spondylitis, we evaluated the diagnostic sensitivity and specificity of the definition for ankylosing spondylitis, by reviewing medical records of 229 patients with ankylosing spondylitis at Gangnam Severance Hospital, a tertiary referral hospital in Korea. The diagnostic sensitivity and specificity were 93.4 and 96.4%, respectively. Baseline comorbidities were defined as follows: hypertension, ICD-10 codes I10–I13 and I15 + prescriptions for anti-hypertensive medications or systolic or diastolic BP ≥ 140 mmHg or ≥ 90 mmHg, respectively; dyslipidemia, ICD-10 code E78 + prescriptions for lipid-lowering medications or serum total cholesterol ≥ 240 mg/dl; stroke, ICD-10 codes I63 or I64 during hospitalization with claims for brain computed tomography or magnetic resonance imaging; chronic kidney disease, estimated glomerular filtration rate (calculated using the Modification of Diet in Renal Disease equation) of < 60 ml/min/1.73m^2^; ischemic heart disease, ICD-10 codes I20–I25; congestive heart failure, ICD-10 code I50; chronic obstructive pulmonary disease (COPD), ICD-10 codes J41–J44; liver cirrhosis, ICD-10 codes K70 or K74; and malignancy, ICD-10 codes C00–C96 (23, 35–40). The outcomes were newly diagnosed depression or anxiety, defined as ICD-10 codes F32–F33 or F40–F41, respectively (41).

### Statistical analysis

Continuous variables are expressed as mean ± standard deviation and categorical variables are expressed as numbers (%). Continuous variables were compared using independent Student’s *t* test and categorical variables were compared using *χ*^2^ test. The incidences of outcomes were expressed as event numbers per 1,000 person-years. The cumulative incidences of events according to the existence of IMIDs were visualized using the Kaplan–Meier curve analysis and compared using log-rank test. Six Cox proportional hazard models were conducted to estimate the hazard ratio (HR) and 95% confidence interval (CI) for the outcomes according to the (i) existence of organ-based IMIDs [gut IMIDs (Crohn’s disease or ulcerative colitis), joint IMIDs (rheumatoid arthritis or ankylosing spondylitis), and skin IMID (psoriasis)]; and (ii) number of IMIDs present (0, 1, and ≥ 2). Model 1 was a univariable model. Model 2 was adjusted for age and sex. Additional adjustment for other potential confounders were made in the subsequent models. The final model (model 6) was adjusted for age, sex, smoking status, alcohol consumption, regular physical activity, income, residence, hypertension, dyslipidemia, body mass index, use of insulin, number of oral hypoglycemic agent ≥ 3, duration of T2DM of ≥ 5 years, chronic kidney disease, stroke, ischemic heart disease, congestive heart failure, COPD, liver cirrhosis, and malignancy. Subgroup analyses were performed by stratifying patients according to several risk factors. All *value of p*s were two-sided, and a *value of p* < 0.05 was considered statistically significant. Statistical analyses were performed using SAS version 9.4 (SAS Institute, Cary, NC, United States).

## Results

### Baseline characteristics

The baseline characteristics of the 1,612,705 patients with T2DM, and the comparison of the characteristics according to the existence of each IMID are reported in Table 1. The mean age was 54.86 ± 12.28 years, and 1,128,324 (69.96%) patients were male. Of the 1,612,705 patients with T2DM, Crohn’s disease, ulcerative colitis, rheumatoid arthritis, ankylosing spondylitis, and psoriasis were present in 93 (0.006%), 549 (0.034%), 30,976 (1.921%), 518 (0.032%), and 13,100 (0.812%) patients, respectively. In comparison with their respective controls, patients with Crohn’s disease and ankylosing spondylitis were younger, whereas those with rheumatoid arthritis and psoriasis were older. The proportion of men was higher in patients with Crohn’s disease, ulcerative colitis, ankylosing spondylitis, and psoriasis, while that of women was higher in patients with rheumatoid arthritis, compared with their respective controls. The proportion of current smokers was lower in patients with ulcerative colitis and rheumatoid arthritis, but higher in those with ankylosing spondylitis and psoriasis, compared with their respective controls. Compared with their respective cohort, the proportion of heavy alcohol drinkers was lower in patients with ulcerative colitis and rheumatoid arthritis, but higher in those with ankylosing spondylitis. With regards to income, the proportion of patients with low income (lowest 25 percentile) was lower in patients with ulcerative colitis and ankylosing spondylitis, but higher in those with rheumatoid arthritis and psoriasis, compared with their respective controls. The existence of baseline comorbidity varied among patients with each IMID. Briefly, compared with their respective controls, hypertension and dyslipidemia were less common, while liver cirrhosis was more common in patients with Crohn’s disease; hypertension and dyslipidemia were less common, while COPD was more common in patients with ulcerative colitis; all comorbidities were more common in patients with rheumatoid arthritis; chronic kidney disease was less common in patients with ankylosing spondylitis; and all comorbidities except chronic kidney disease were more common in patients with psoriasis.

**Table 1:** Baseline characteristics of the patients with type 2 diabetes according to the presence of immune-mediated inflammatory diseases.

	Total population	No Crohn’s disease	Crohn’s disease	*Value of p*	No ulcerative colitis	Ulcerative colitis	*Value of p*	No rheumatoid arthritis	Rheumatoid arthritis	*Value of p*	No ankylosing spondylitis	Ankylosing spondylitis	*Value of p*	No psoriasis	Psoriasis	*Value of p*
Number of patients	1,612,705	1,612,612	93		1,612,156	549		1,581,729	30,976		1,612,187	518		1,599,605	13,100	
Age, years, Mean ± SD	54.86 ± 12.28	54.86 ± 12.28	49.68 ± 13.94	<0.0001	54.86 ± 12.28	55.75 ± 11.98	0.0909	54.76 ± 12.28	59.87 ± 11.13	<0.0001	54.86 ± 12.28	48.52 ± 12.32	<0.0001	54.85 ± 12.28	56.67 ± 11.76	<0.0001
Age, years, *n* (%)				<0.0001			0.1692			<0.0001			<0.0001			<0.0001
20–40	170,501 (10.57)	170,475 (10.57)	26 (27.96)		170,454 (10.57)	47 (8.56)		169,311 (10.7)	1,190 (3.84)		170,362 (10.57)	139 (26.83)		169,521 (10.6)	980 (7.48)	
40–65	1,076,123 (66.73)	1,076,074 (66.73)	49 (52.69)		1,075,759 (66.73)	364 (66.3)		1,057,317 (66.85)	18,806 (60.71)		1,075,809 (66.73)	314 (60.62)		1,067,457 (66.73)	8,666 (66.15)	
65≤	366,081 (22.7)	366,063 (22.7)	18 (19.35)		365,943 (22.7)	138 (25.14)		355,101 (22.45)	10,980 (35.45)		366,016 (22.7)	65 (12.55)		362,627 (22.67)	3,454 (26.37)	
Male sex, *n* (%)	1,128,324 (69.96)	1,128,245 (69.96)	79 (84.95)	0.0016	1,127,891 (69.96)	433 (78.87)	<0.0001	1,113,425 (70.39)	14,899 (48.1)	<0.0001	1,127,849 (69.96)	475 (91.7)	<0.0001	1,118,357 (69.91)	9,967 (76.08)	<0.0001
BMI, kg/m^2^, Mean ± SD	25.12 ± 3.39	25.12 ± 3.39	23.7 ± 3.39	<0.0001	25.12 ± 3.39	24.43 ± 3.05	<0.0001	25.12 ± 3.39	25.15 ± 3.42	0.2579	25.12 ± 3.39	25.52 ± 3.94	0.0071	25.12 ± 3.39	25.12 ± 3.34	0.8604
Smoking, *n* (%)				0.1384			<0.0001			<0.0001			<0.0001			<0.0001
Non-smoker	777,342 (48.2)	777,302 (48.2)	40 (43.01)		777,097 (48.2)	245 (44.63)		757,359 (47.88)	19,983 (64.51)		777,202 (48.21)	140 (27.03)		771,925 (48.26)	5,417 (41.35)	
Ex-smoker	333,529 (20.68)	333,502 (20.68)	27 (29.03)		333,297 (20.67)	232 (42.26)		328,236 (20.75)	5,293 (17.09)		333,382 (20.68)	147 (28.38)		330,204 (20.64)	3,325 (25.38)	
Current smoker	501,834 (31.12)	501,808 (31.12)	26 (27.96)		501,762 (31.12)	72 (13.11)		496,134 (31.37)	5,700 (18.4)		501,603 (31.11)	231 (44.59)		497,476 (31.1)	4,358 (33.27)	
Alcohol consumption, *n* (%)				0.1278			<0.0001			<0.0001			0.02			0.1057
None	790,465 (49.01)	790,414 (49.01)	51 (54.84)		790,137 (49.01)	328 (59.74)		769,961 (48.68)	20,504 (66.19)		790,241 (49.02)	224 (43.24)		783,924 (49.01)	6,541 (49.93)	
Mild drinker	627,568 (38.91)	627,531 (38.91)	37 (39.78)		627,387 (38.92)	181 (32.97)		619,324 (39.15)	8,244 (26.61)		627,337 (38.91)	231 (44.59)		622,570 (38.92)	4,998 (38.15)	
Heavy drinker	194,672 (12.07)	194,667 (12.07)	5 (5.38)		194,632 (12.07)	40 (7.29)		192,444 (12.17)	2,228 (7.19)		194,609 (12.07)	63 (12.16)		193,111 (12.07)	1,561 (11.92)	
Regular physical activity, *n* (%)	341,698 (21.19)	341,681 (21.19)	17 (18.28)	0.4925	341,573 (21.19)	125 (22.77)	0.3646	335,501 (21.21)	6,197 (20.01)	<0.0001	341,608 (21.19)	90 (17.37)	0.0337	338,870 (21.18)	2,828 (21.59)	0.2607
Low income, *n* (%)	367,311 (22.78)	367,294 (22.78)	17 (18.28)	0.3011	367,210 (22.78)	101 (18.4)	0.0144	360,082 (22.77)	7,229 (23.34)	0.0174	367,230 (22.78)	81 (15.64)	0.0001	364,249 (22.77)	3,062 (23.37)	0.1013
Rural residence, *n* (%)	884,581 (54.85)	884,532 (54.85)	49 (52.69)	0.6751	884,297 (54.85)	284 (51.73)	0.1417	867,223 (54.83)	17,358 (56.04)	<0.0001	884,292 (54.85)	289 (55.79)	0.667	877,296 (54.84)	7,285 (55.61)	0.0793
Hypertension, *n* (%)	842,878 (52.26)	842,841 (52.27)	37 (39.78)	0.016	842,643 (52.27)	235 (42.81)	<0.0001	824,041 (52.1)	18,837 (60.81)	<0.0001	842,624 (52.27)	254 (49.03)	0.141	835,484 (52.23)	7,394 (56.44)	<0.0001
Dyslipidemia, *n* (%)	608,471 (37.73)	608,450 (37.73)	21 (22.58)	0.0026	608,292 (37.73)	179 (32.6)	0.0132	594,124 (37.56)	14,347 (46.32)	<0.0001	608,283 (37.73)	188 (36.29)	0.4999	602,843 (37.69)	5,628 (42.96)	<0.0001
CKD, *n* (%)	146,156 (9.06)	146,144 (9.06)	12 (12.9)	0.197	146,119 (9.06)	37 (6.74)	0.0579	142,129 (8.99)	4,027 (13)	<0.0001	146,124 (9.06)	32 (6.18)	0.0222	144,951 (9.06)	1,205 (9.2)	0.587
Stroke, *n* (%)	44,730 (2.77)	44,728 (2.77)	2 (2.15)	0.7144	44,712 (2.77)	18 (3.28)	0.471	43,242 (2.73)	1,488 (4.8)	<0.0001	44,720 (2.77)	10 (1.93)	0.2425	44,240 (2.77)	490 (3.74)	<0.0001
IHD, *n* (%)	142,197 (8.82)	142,189 (8.82)	8 (8.6)	0.9417	142,143 (8.82)	54 (9.84)	0.3998	137,667 (8.7)	4,530 (14.62)	<0.0001	142,156 (8.82)	41 (7.92)	0.4689	140,565 (8.79)	1,632 (12.46)	<0.0001
CHF, *n* (%)	19,784 (1.23)	19,784 (1.23)	0 (0)	0.2825	19,779 (1.23)	5 (0.91)	0.5011	19,116 (1.21)	668 (2.16)	<0.0001	19,776 (1.23)	8 (1.54)	0.5113	19,572 (1.22)	212 (1.62)	<0.0001
COPD, *n* (%)	103,896 (6.44)	103,892 (6.44)	4 (4.3)	0.4003	103,847 (6.44)	49 (8.93)	0.0178	100,008 (6.32)	3,888 (12.55)	<0.0001	103,852 (6.44)	44 (8.49)	0.0571	102,676 (6.42)	1,220 (9.31)	<0.0001
LC, *n* (%)	11,713 (0.73)	11,710 (0.73)	3 (3.23)	0.0045	11,706 (0.73)	7 (1.28)	0.1299	11,434 (0.72)	279 (0.9)	0.0003	11,711 (0.73)	2 (0.39)	0.3618	11,574 (0.72)	139 (1.06)	<0.0001
Malignancy, *n* (%)	38,535 (2.39)	38,533 (2.39)	2 (2.15)	0.8801	38,509 (2.39)	26 (4.74)	0.0003	37,554 (2.37)	981 (3.17)	<0.0001	38,523 (2.39)	12 (2.32)	0.9135	38,135 (2.38)	400 (3.05)	<0.0001
Use of insulin, *n* (%)	112,119 (6.95)	112,106 (6.95)	13 (13.98)	0.0077	112,054 (6.95)	65 (11.84)	<0.0001	108,128 (6.84)	3,991 (12.88)	<0.0001	112,072 (6.95)	47 (9.07)	0.0576	110,825 (6.93)	1,294 (9.88)	<0.0001
Number of oral hypoglycemic agents used ≥3, *n* (%)	210,037 (13.02)	210,023 (13.02)	14 (15.05)	0.5608	209,974 (13.02)	63 (11.48)	0.2809	204,801 (12.95)	5,236 (16.9)	<0.0001	209,981 (13.02)	56 (10.81)	0.1344	208,082 (13.01)	1955 (14.92)	<0.0001
Duration of type 2 diabetes ≥5 years, *n* (%)	430,856 (26.72)	430,831 (26.72)	25 (26.88)	0.9712	430,691 (26.72)	165 (30.05)	0.0771	420,406 (26.58)	10,450 (33.74)	<0.0001	430,740 (26.72)	116 (22.39)	0.0262	427,023 (26.7)	3,833 (29.26)	<0.0001

BMI, body mass index; CKD, chronic kidney disease; IHD, ischemic heart disease; CHF, congestive heart failure; COPD, chronic obstructive pulmonary disease; LC, liver cirrhosis; SD, standard deviation.

### Incidence and risk of depression according to the presence of IMIDs

**Figure 2:**
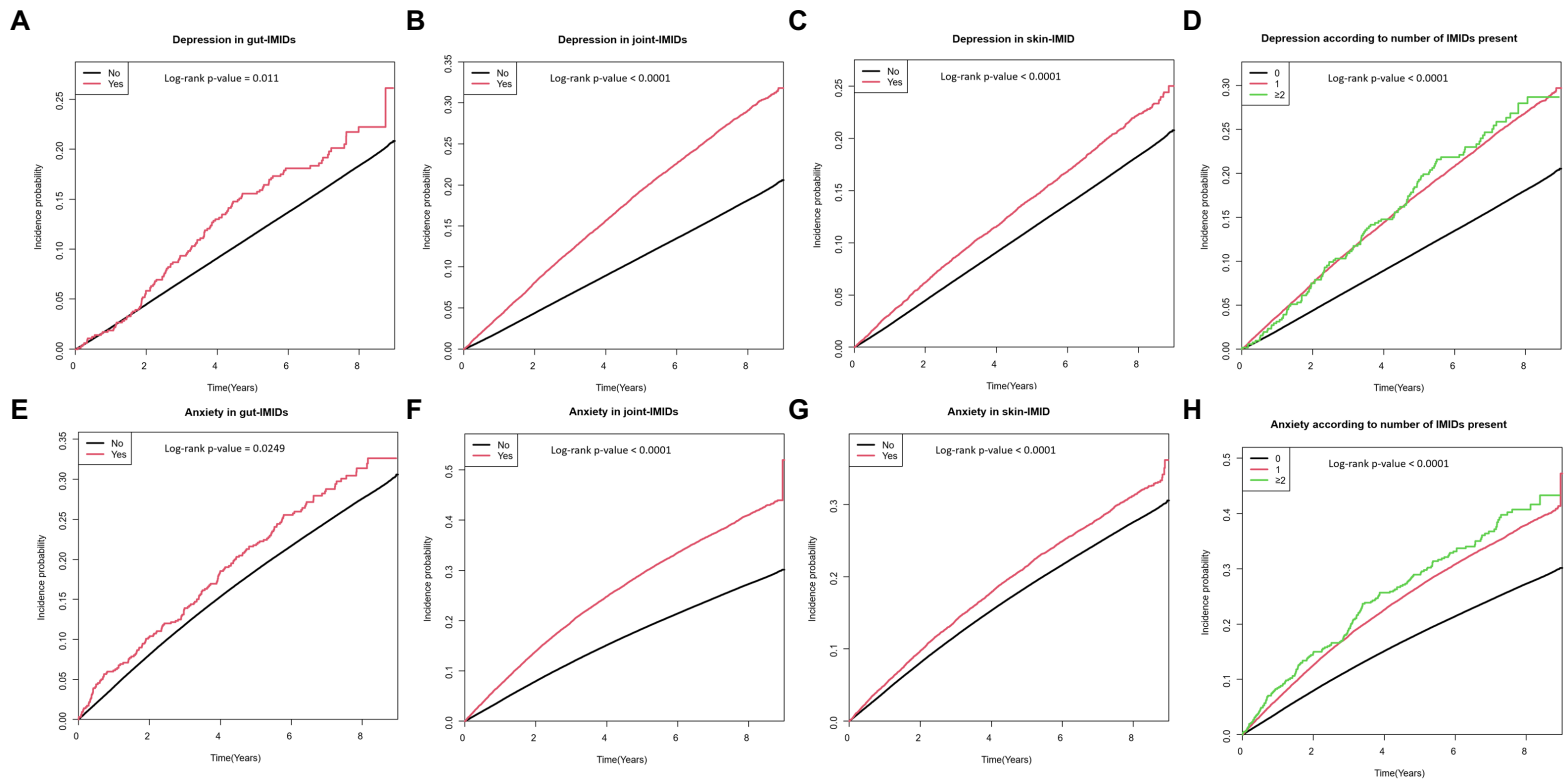
Cumulative incidence of depression in patients with **(A)** gut IMIDs, **(B)** joint IMIDs, **(C)** skin IMID, and **(D)** different numbers of IMIDs (0, 1, and ≥ 2); and cumulative incidence of anxiety in patients with **(E)** gut IMIDs, **(F)** joint IMIDs, **(G)** skin IMID, and **(H)** different numbers of IMIDs (0, 1, and ≥ 2). IMIDs, immune-mediated inflammatory disease.

**Figure 3:**
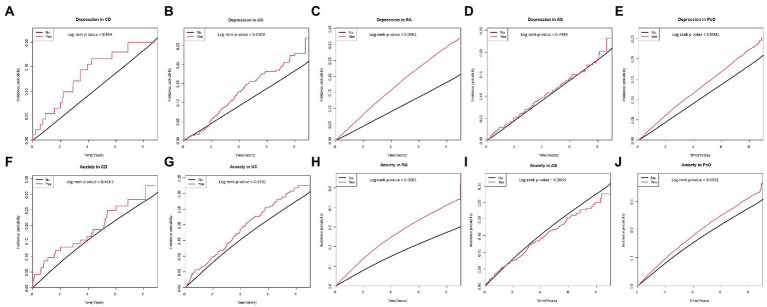
Cumulative incidence of depression in patients with **(A)** CD, **(B)** UC, **(C)** RA, **(D)** AS, and **(E)** PsO; and cumulative incidence of anxiety in patients with **(F)** CD, **(G)** UC, **(H)** RA, **(I)** AS, and **(J)** PsO. CD, crohn’s disease; UC, ulcerative colitis; RA, rheumatoid arthritis; AS, ankylosing spondylitis; PsO, psoriasis.

Table 2 shows the incidence rates and HRs of depression, according to the existence of IMIDs. The incidence rates of depression in patients with T2DM with gut IMIDs, joint IMIDs, and skin IMID were 31.32, 42.98, and 31.23 per 1,000 person-years, respectively. The incidence rates of depression stratified by specific diagnoses are summarized in Supplementary Table 1. The cumulative incidence of depression according to the existence of organ-based IMIDs is shown in Figures 2A–C. The univariable Cox proportional hazard model (model 1) revealed that existence of gut IMIDs, joint IMIDs, and skin IMID, respectively, was significantly associated with a higher risk of depression. After adjusting for potential confounders in model 6, existence of gut IMIDs (adjusted HR: 1.284 [95% CI: 1.077–1.530]), joint IMIDs (adjusted HR: 1.335 [95% CI: 1.306–1.365]), and skin IMID (adjusted HR: 1.183 [95% CI: 1.138–1.230]) was significantly associated with a higher risk of depression, respectively. According to the number of IMIDs present, patients with one IMID (adjusted HR: 1.296 [95% CI: 1.271–1.322]) had a significantly higher risk of depression than those without any IMIDs, and patients with ≥ 2 IMIDs (adjusted HR: 1.415 [95% CI: 1.187–1.687]) also had a significantly higher risk of depression than those without any IMIDs, with a larger effect size. When incident anxiety was additionally adjusted (model 7), the associations observed in model 6 remained statistically significant. The cumulative incidence and HR of depression, according to the existence of individual IMIDs, are shown in Figures 3A–E and Table 3.

**Table 2:** Risk of depression according to the presence of immune-mediated inflammatory diseases.

	*N*	Depression	Duration	IR per 1,000	Model 1	Model 2	Model 3	Model 4	Model 5	Model 6	Model 7
Gut IMIDs (Crohn’s disease or ulcerative colitis)											
No	1,612,067	258,018	10322330.67	24.9961	1(Ref.)	1(Ref.)	1(Ref.)	1(Ref.)	1(Ref.)	1(Ref.)	1 (ref.)
Yes	638	125	3990.72	31.3227	1.255 (1.053,1.496)	1.29 (1.082,1.537)	1.32 (1.108,1.573)	1.324 (1.111,1.578)	1.29 (1.083,1.538)	1.284 (1.077,1.53)	1.197 (1.005, 1.427)
Joint IMIDs (rheumatoid arthritis or ankylosing spondylitis)											
No	1,581,324	250,124	10139725.04	24.6677	1(Ref.)	1(Ref.)	1(Ref.)	1(Ref.)	1(Ref.)	1(Ref.)	1 (ref.)
Yes	31,381	8,019	186596.34	42.9751	1.748 (1.709,1.787)	1.396 (1.365,1.428)	1.396 (1.365,1.428)	1.392 (1.361,1.423)	1.36 (1.33,1.391)	1.335 (1.306,1.365)	1.224 (1.197, 1.251)
Skin IMID (psoriasis)											
No	1,599,605	255,597	10244787.62	24.949	1(Ref.)	1(Ref.)	1(Ref.)	1(Ref.)	1(Ref.)	1(Ref.)	1 (ref.)
Yes	13,100	2,546	81533.77	31.2263	1.253 (1.205,1.303)	1.217 (1.17,1.265)	1.214 (1.167,1.262)	1.208 (1.162,1.257)	1.196 (1.15,1.244)	1.183 (1.138,1.23)	1.15 (1.106, 1.195)
Number of IMIDs											
0	1,567,988	247,560	10056564.14	24.6168	1(Ref.)	1(Ref.)	1(Ref.)	1(Ref.)	1(Ref.)	1(Ref.)	1 (ref.)
1	44,206	10,459	266715.84	39.214	1.597 (1.566,1.628)	1.348 (1.322,1.375)	1.348 (1.322,1.375)	1.343 (1.317,1.37)	1.318 (1.292,1.344)	1.296 (1.271,1.322)	1.207 (1.183, 1.231)
≥2	511	124	3041.41	40.7706	1.663 (1.395,1.983)	1.532 (1.284,1.826)	1.523 (1.277,1.816)	1.507 (1.263,1.797)	1.453 (1.218,1.733)	1.415 (1.187,1.687)	1.259 (1.056, 1.501)

Model 1 adjusted for none of the covariates (univariable analysis).

Model 2 adjusted for age and sex.

Model 3 adjusted for age, sex, smoking, alcohol consumption, regular physical activity, income, and residence.

Model 4 adjusted for age, sex, smoking, alcohol consumption, regular physical activity, income, residence, hypertension, dyslipidemia, and BMI.

Model 5 adjusted for age, sex, smoking, alcohol consumption, regular physical activity, income, residence, hypertension, dyslipidemia, BMI, use of insulin, number of oral hypoglycemic agent ≥ 3, and duration of type 2 diabetes ≥ 5 years.

Model 6 adjusted for age, sex, smoking, alcohol consumption, regular physical activity, income, residence, hypertension, dyslipidemia, BMI, use of insulin, number of oral hypoglycemic agent ≥ 3, duration of type 2 diabetes ≥ 5 years, CKD, stroke, IHD, CHF, COPD, LC, and malignancy.

IR, incidence rate; IMIDs, immune-mediated inflammatory diseases; BMI, body mass index; CKD, chronic kidney disease; IHD, ischemic heart disease; CHF, congestive heart failure; COPD, chronic obstructive pulmonary disease; LC, liver cirrhosis.

**Table 3:** Risk of depression according to the presence of individual immune-mediated inflammatory diseases.

	*N*	Depression	Duration	IR per 1,000	Model 1	Model 2	Model 3	Model 4	Model 5	Model 6	Model 7
Crohn’s disease											
No	1,612,612	258,126	10325763.77	24.9982	1(Ref.)	1(Ref.)	1(Ref.)	1(Ref.)	1(Ref.)	1(Ref.)	1 (ref.)
Yes	93	17	557.62	30.4868	1.251 (0.782,2.002)	1.515 (0.942,2.436)	1.548 (0.963,2.489)	1.551 (0.965,2.494)	1.485 (0.923,2.389)	1.477 (0.918,2.376)	1.481 (0.92, 2.382)
Ulcerative colitis											
No	1,612,156	258,033	10322869.77	24.9962	1(Ref.)	1(Ref.)	1(Ref.)	1(Ref.)	1(Ref.)	1(Ref.)	1 (ref.)
Yes	549	110	3451.62	31.8691	1.277 (1.059,1.539)	1.273 (1.056,1.534)	1.303 (1.08,1.57)	1.307 (1.084,1.575)	1.275 (1.058,1.537)	1.267 (1.051,1.528)	1.173 (0.973, 1.414)
Rheumatoid arthritis											
No	1,581,729	250,193	10142313.25	24.6682	1(Ref.)	1(Ref.)	1(Ref.)	1(Ref.)	1(Ref.)	1(Ref.)	1 (ref.)
Yes	30,976	7,950	184008.13	43.2046	1.757 (1.718,1.797)	1.396 (1.365,1.427)	1.396 (1.365,1.427)	1.391 (1.36,1.423)	1.36 (1.33,1.391)	1.335 (1.305,1.365)	1.223 (1.196, 1.25)
Ankylosing spondylitis											
No	1,612,187	258,058	10323036.39	24.9983	1(Ref.)	1(Ref.)	1(Ref.)	1(Ref.)	1(Ref.)	1(Ref.)	1 (ref.)
Yes	518	85	3,285	25.8752	1.037 (0.838,1.282)	1.391 (1.124,1.72)	1.395 (1.128,1.726)	1.394 (1.127,1.725)	1.37 (1.108,1.695)	1.353 (1.093,1.673)	1.286 (1.04, 1.591)
Psoriasis											
No	1,599,605	255,597	10244787.62	24.949	1(Ref.)	1(Ref.)	1(Ref.)	1(Ref.)	1(Ref.)	1(Ref.)	1 (ref.)
Yes	13,100	2,546	81533.77	31.2263	1.253 (1.205,1.303)	1.217 (1.17,1.265)	1.214 (1.167,1.262)	1.208 (1.162,1.257)	1.196 (1.15,1.244)	1.183 (1.138,1.23)	1.15 (1.106, 1.195)

Model 1 adjusted for none of the covariates (univariable analysis).

Model 2 adjusted for age and sex.

Model 3 adjusted for age, sex, smoking, alcohol consumption, regular physical activity, income, and residence.

Model 4 adjusted for age, sex, smoking, alcohol consumption, regular physical activity, income, residence, hypertension, dyslipidemia, and BMI.

Model 5 adjusted for age, sex, smoking, alcohol consumption, regular physical activity, income, residence, hypertension, dyslipidemia, BMI, use of insulin, number of oral hypoglycemic agent ≥ 3, and duration of type 2 diabetes ≥ 5 years.

Model 6 adjusted for age, sex, smoking, alcohol consumption, regular physical activity, income, residence, hypertension, dyslipidemia, BMI, use of insulin, number of oral hypoglycemic agent ≥ 3, duration of type 2 diabetes ≥ 5 years, CKD, stroke, IHD, CHF, COPD, LC, and malignancy.

IR, incidence rate; BMI, body mass index; CKD, chronic kidney disease; IHD, ischemic heart disease; CHF, congestive heart failure; COPD, chronic obstructive pulmonary disease; LC, liver cirrhosis.

### Incidence and risk of anxiety according to the presence of IMIDs

The incidence rates and HRs of anxiety, according to the existence of IMIDs are summarized in Table 4. The incidence rates of anxiety in patients with T2DM with gut IMIDs, joint IMIDs, and skin IMID were 48.09, 67.82, and 47.51 per 1,000 person-years, respectively. The incidence rates of anxiety stratified by specific diagnoses are reported in Supplementary Table 2. The cumulative incidence of anxiety according to the existence of organ-based IMIDs is depicted in Figures 2E–G. The univariable Cox proportional hazard model (model 1) revealed that existence of gut IMIDs, joint IMIDs, and skin IMID, respectively, was significantly associated with a higher risk of anxiety. In the final model (model 6) adjusted for potential confounders, existence of gut IMIDs (adjusted HR: 1.223 [95% CI: 1.057–1.416]), joint IMIDs (adjusted HR: 1.311 [95% CI: 1.287–1.335]), and skin IMID (adjusted HR: 1.126 [95% CI: 1.090–1.163]) was significantly associated with a higher risk of anxiety, respectively. According to the number of IMIDs present, patients with one IMID (adjusted HR: 1.259 [95% CI: 1.238–1.279]) had a significantly higher risk of anxiety than those without any IMIDs, and patients with ≥ 2 IMIDs (adjusted HR: 1.491 [95% CI: 1.291–1.722]) also had a significantly higher risk of anxiety than those without any IMIDs, with a larger effect size. When incident depression was additionally adjusted (model 7), similar associations were observed as in model 6. Existence of gut IMIDs (adjusted HR: 1.14 [95% CI: 0.985–1.319]) showed a trend of being associated with a higher risk of anxiety, and existence of joint IMIDs (adjusted HR: 1.224 [95% CI: 1.201–1.247]), and skin IMID (adjusted HR: 1.094 [95% CI: 1.059–1.131]) was significantly associated with a higher risk of anxiety. The cumulative incidence and HR of anxiety, according to the existence of individual IMIDs, are shown in Figures 3F–J and Table 5.

**Table 4:** Risk of anxiety according to the presence of immune-mediated inflammatory diseases.

	*N*	Anxiety	Duration	IR per 1,000	Model1	Model2	Model3	Model4	Model5	Model6	Model7
Gut IMIDs (Crohn’s disease or ulcerative colitis)											
No	1,612,067	395,169	9714870.15	40.6767	1(Ref.)	1(Ref.)	1(Ref.)	1(Ref.)	1(Ref.)	1(Ref.)	1 (ref.)
Yes	638	180	3742.99	48.0899	1.199 (1.037,1.387)	1.233 (1.066,1.427)	1.245 (1.076,1.441)	1.249 (1.079,1.445)	1.229 (1.062,1.423)	1.223 (1.057,1.416)	1.14 (0.985, 1.319)
Joint IMIDs (rheumatoid arthritis or ankylosing spondylitis)											
No	1,581,324	383,841	9548922.79	40.1973	1(Ref.)	1(Ref.)	1(Ref.)	1(Ref.)	1(Ref.)	1(Ref.)	1 (ref.)
Yes	31,381	11,508	169690.34	67.8176	1.684 (1.653,1.715)	1.358 (1.333,1.384)	1.358 (1.333,1.383)	1.354 (1.329,1.38)	1.336 (1.312,1.362)	1.311 (1.287,1.335)	1.224 (1.201, 1.247)
Skin IMID (psoriasis)											
No	1,599,605	391,710	9642017.01	40.6253	1(Ref.)	1(Ref.)	1(Ref.)	1(Ref.)	1(Ref.)	1(Ref.)	1 (ref.)
Yes	13,100	3,639	76596.13	47.5089	1.169 (1.131,1.208)	1.151 (1.114,1.189)	1.15 (1.113,1.188)	1.147 (1.11,1.185)	1.139 (1.102,1.177)	1.126 (1.09,1.163)	1.094 (1.059, 1.131)
Number of IMIDs											
0	1,567,988	380,184	9470662.27	40.1433	1(Ref.)	1(Ref.)	1(Ref.)	1(Ref.)	1(Ref.)	1(Ref.)	1 (ref.)
1	44,206	14,980	245212.66	61.0898	1.519 (1.495,1.544)	1.299 (1.277,1.32)	1.298 (1.277,1.32)	1.295 (1.274,1.316)	1.28 (1.259,1.301)	1.259 (1.238,1.279)	1.187 (1.168, 1.207)
≥2	511	185	2738.21	67.5623	1.679 (1.454,1.94)	1.592 (1.378,1.839)	1.588 (1.375,1.835)	1.576 (1.364,1.82)	1.538 (1.331,1.776)	1.491 (1.291,1.722)	1.364 (1.181, 1.576)

Model 1 adjusted for none of the covariates (univariable analysis).

Model 2 adjusted for age and sex.

Model 3 adjusted for age, sex, smoking, alcohol consumption, regular physical activity, income, and residence.

Model 4 adjusted for age, sex, smoking, alcohol consumption, regular physical activity, income, residence, hypertension, dyslipidemia, and BMI.

Model 5 adjusted for age, sex, smoking, alcohol consumption, regular physical activity, income, residence, hypertension, dyslipidemia, BMI, use of insulin, number of oral hypoglycemic agent ≥ 3, and duration of type 2 diabetes ≥ 5 years.

Model 6 adjusted for age, sex, smoking, alcohol consumption, regular physical activity, income, residence, hypertension, dyslipidemia, BMI, use of insulin, number of oral hypoglycemic agent ≥ 3, duration of type 2 diabetes ≥ 5 years, CKD, stroke, IHD, CHF, COPD, LC, and malignancy.

IR, incidence rate; IMIDs, immune-mediated inflammatory diseases; BMI, body mass index; CKD, chronic kidney disease; IHD, ischemic heart disease; CHF, congestive heart failure; COPD, chronic obstructive pulmonary disease; LC, liver cirrhosis.

**Table 5:** Risk of anxiety according to the presence of individual immune-mediated inflammatory diseases.

	*N*	Anxiety	Duration	IR per 1,000	Model1	Model2	Model3	Model4	Model5	Model6	Model7
Crohn’s disease											
No	1,612,612	395,324	9718082.02	40.6792	1(Ref.)	1(Ref.)	1(Ref.)	1(Ref.)	1(Ref.)	1(Ref.)	1 (ref.)
Yes	93	25	531.12	47.0705	1.17 (0.792,1.727)	1.363 (0.921,2.016)	1.375 (0.929,2.034)	1.371 (0.927,2.029)	1.329 (0.898,1.967)	1.332 (0.9,1.972)	1.202 (0.812, 1.779)
Ulcerative colitis											
No	1,612,156	395,193	9715380.63	40.677	1(Ref.)	1(Ref.)	1(Ref.)	1(Ref.)	1(Ref.)	1(Ref.)	1 (ref.)
Yes	549	156	3232.51	48.2597	1.204 (1.031,1.407)	1.208 (1.033,1.414)	1.22 (1.043,1.428)	1.225 (1.047,1.433)	1.208 (1.033,1.414)	1.2 (1.026,1.404)	1.118 (0.956, 1.308)
Rheumatoid arthritis											
No	1,581,729	383,933	9551378.85	40.1966	1(Ref.)	1(Ref.)	1(Ref.)	1(Ref.)	1(Ref.)	1(Ref.)	1 (ref.)
Yes	30,976	11,416	167234.29	68.2635	1.695 (1.664,1.727)	1.36 (1.335,1.385)	1.359 (1.334,1.385)	1.355 (1.33,1.381)	1.338 (1.313,1.363)	1.312 (1.287,1.337)	1.225 (1.202, 1.248)
Ankylosing spondylitis											
No	1,612,187	395,234	9715477.15	40.6809	1(Ref.)	1(Ref.)	1(Ref.)	1(Ref.)	1(Ref.)	1(Ref.)	1 (ref.)
Yes	518	115	3135.99	36.6711	0.906 (0.755,1.087)	1.181 (0.984,1.418)	1.185 (0.987,1.423)	1.185 (0.987,1.423)	1.171 (0.975,1.406)	1.16 (0.966,1.393)	1.067 (0.889, 1.281)
Psoriasis											
No	1,599,605	391,710	9642017.01	40.6253	1(Ref.)	1(Ref.)	1(Ref.)	1(Ref.)	1(Ref.)	1(Ref.)	1 (ref.)
Yes	13,100	3,639	76596.13	47.5089	1.169 (1.131,1.208)	1.151 (1.114,1.189)	1.15 (1.113,1.188)	1.147 (1.11,1.185)	1.139 (1.102,1.177)	1.126 (1.09,1.163)	1.094 (1.059, 1.131)

Model 1 adjusted for none of the covariates (univariable analysis).

Model 2 adjusted for age and sex.

Model 3 adjusted for age, sex, smoking, alcohol consumption, regular physical activity, income, and residence.

Model 4 adjusted for age, sex, smoking, alcohol consumption, regular physical activity, income, residence, hypertension, dyslipidemia, and BMI.

Model 5 adjusted for age, sex, smoking, alcohol consumption, regular physical activity, income, residence, hypertension, dyslipidemia, BMI, use of insulin, number of oral hypoglycemic agent ≥ 3, and duration of type 2 diabetes ≥ 5 years.

Model 6 adjusted for age, sex, smoking, alcohol consumption, regular physical activity, income, residence, hypertension, dyslipidemia, BMI, use of insulin, number of oral hypoglycemic agent ≥ 3, duration of type 2 diabetes ≥ 5 years, CKD, stroke, IHD, CHF, COPD, LC, and malignancy.

IR, incidence rate; BMI, body mass index; CKD, chronic kidney disease; IHD, ischemic heart disease; CHF, congestive heart failure; COPD, chronic obstructive pulmonary disease; LC, liver cirrhosis.

### Incidence and risk of developing both depression and anxiety according to the presence of IMIDs

As depression and anxiety often co-occur, we additionally analyzed the association between presence of IMIDs and occurrence of both depression and anxiety (Table 6). Similar associations were observed as in the analyses where depression and anxiety were analyzed separately. In model 6, existence of joint IMIDs (adjusted HR: 1.266 [95% CI: 1.151–1.394]), and skin IMID (adjusted HR: 1.252 [95% CI: 1.075–1.459]) was significantly associated with a higher risk of occurrence of both depression and anxiety. Existence of gut IMIDs (adjusted HR: 1.5 [95% CI: 0.78–2.883]) showed a trend of being associated with a higher risk of occurrence of both depression and anxiety. According to the number of IMIDs present, patients with one IMID (adjusted HR: 1.247 [95% CI: 1.148–1.354]) had a significantly higher risk of occurrence of both depression and anxiety than those without any IMIDs, and patients with ≥ 2 IMIDs (adjusted HR: 2.234 [95% CI: 1.24–4.025]) also had a significantly higher risk of occurrence of both depression and anxiety than those without any IMIDs, with a larger effect size.

**Table 6:** Risk of developing both depression and anxiety according to the presence of immune-mediated inflammatory diseases.

	*N*	Depression and anxiety	Duration	IR (per 1,000)	Model 1	Model 2	Model 3	Model 4	Model 5	Model 6
Gut IMIDs										
No	1,612,067	16,699	9233754.08	1.80847	1 (ref.)	1 (ref.)	1 (ref.)	1 (ref.)	1 (ref.)	1 (ref.)
Yes	638	9	3481.16	2.58535	1.439 (0.749, 2.764)	1.502 (0.782, 2.885)	1.543 (0.803, 2.963)	1.539 (0.801, 2.957)	1.505 (0.783, 2.894)	1.5 (0.78, 2.883)
Joint IMIDs										
No	1,581,324	16,274	9081815.75	1.79193	1 (ref.)	1 (ref.)	1 (ref.)	1 (ref.)	1 (ref.)	1 (ref.)
Yes	31,381	434	155419.49	2.79244	1.576 (1.433, 1.734)	1.314 (1.194, 1.446)	1.314 (1.195, 1.446)	1.312 (1.192, 1.444)	1.289 (1.172, 1.419)	1.266 (1.151, 1.394)
Skin IMID										
No	1,599,605	16,541	9165309.53	1.80474	1 (ref.)	1 (ref.)	1 (ref.)	1 (ref.)	1 (ref.)	1 (ref.)
Yes	13,100	167	71925.72	2.32184	1.29 (1.108, 1.502)	1.281 (1.1, 1.492)	1.278 (1.097, 1.488)	1.275 (1.095, 1.485)	1.265 (1.087, 1.474)	1.252 (1.075, 1.459)
Number of IMIDs										
0	1,567,988	16,109	9008330.97	1.78823	1 (ref.)	1 (ref.)	1 (ref.)	1 (ref.)	1 (ref.)	1 (ref.)
1	44,206	588	226384.36	2.59735	1.465 (1.349, 1.59)	1.287 (1.185, 1.398)	1.287 (1.185, 1.398)	1.284 (1.183, 1.395)	1.266 (1.166, 1.375)	1.247 (1.148, 1.354)
≥2	511	11	2519.92	4.36523	2.489 (1.381, 4.485)	2.398 (1.331, 4.322)	2.39 (1.326, 4.307)	2.376 (1.319, 4.282)	2.304 (1.279, 4.152)	2.234 (1.24, 4.025)
Crohn’s disease										
No	1,612,612	16,706	9236738.98	1.80865	1 (ref.)	1 (ref.)	1 (ref.)	1 (ref.)	1 (ref.)	1 (ref.)
Yes	93	2	496.27	4.03008	2.243 (0.562, 8.948)	2.641 (0.662, 10.538)	2.708 (0.679, 10.801)	2.686 (0.673, 10.717)	2.574 (0.645, 10.265)	2.586 (0.648, 10.315)
Ulcerative colitis										
No	1,612,156	16,701	9234234.36	1.8086	1 (ref.)	1 (ref.)	1 (ref.)	1 (ref.)	1 (ref.)	1 (ref.)
Yes	549	7	3000.88	2.33265	1.296 (0.618, 2.719)	1.325 (0.632, 2.779)	1.361 (0.649, 2.855)	1.359 (0.648, 2.851)	1.336 (0.637, 2.803)	1.328 (0.633, 2.787)
Rheumatoid arthritis										
No	1,581,729	16,285	9084156.05	1.79268	1 (ref.)	1 (ref.)	1 (ref.)	1 (ref.)	1 (ref.)	1 (ref.)
Yes	30,976	423	153079.19	2.76328	1.559 (1.416, 1.717)	1.293 (1.174, 1.425)	1.294 (1.174, 1.425)	1.291 (1.172, 1.422)	1.269 (1.152, 1.398)	1.246 (1.131, 1.373)
Ankylosing spondylitis										
No	1,612,187	16,697	9234275.8	1.80815	1 (ref.)	1 (ref.)	1 (ref.)	1 (ref.)	1 (ref.)	1 (ref.)
Yes	518	11	2959.45	3.71691	2.059 (1.141, 3.718)	2.581 (1.429, 4.66)	2.596 (1.438, 4.688)	2.602 (1.441, 4.698)	2.57 (1.423, 4.64)	2.549 (1.412, 4.603)
Psoriasis										
No	1,599,605	16,541	9165309.53	1.80474	1 (ref.)	1 (ref.)	1 (ref.)	1 (ref.)	1 (ref.)	1 (ref.)
Yes	13,100	167	71925.72	2.32184	1.29 (1.108, 1.502)	1.281 (1.1, 1.492)	1.278 (1.097, 1.488)	1.275 (1.095, 1.485)	1.265 (1.087, 1.474)	1.252 (1.075, 1.459)

Model 1 adjusted for none of the covariates (univariable analysis).

Model 2 adjusted for age and sex.

Model 3 adjusted for age, sex, smoking, alcohol consumption, regular physical activity, income, and residence.

Model 4 adjusted for age, sex, smoking, alcohol consumption, regular physical activity, income, residence, hypertension, dyslipidemia, and BMI.

Model 5 adjusted for age, sex, smoking, alcohol consumption, regular physical activity, income, residence, hypertension, dyslipidemia, BMI, use of insulin, number of oral hypoglycemic agent ≥ 3, and duration of type 2 diabetes ≥ 5 years.

Model 6 adjusted for age, sex, smoking, alcohol consumption, regular physical activity, income, residence, hypertension, dyslipidemia, BMI, use of insulin, number of oral hypoglycemic agent ≥ 3, duration of type 2 diabetes ≥ 5 years, CKD, stroke, IHD, CHF, COPD, LC, and malignancy.

IR, incidence rate; IMIDs, immune-mediated inflammatory diseases; BMI, body mass index; CKD, chronic kidney disease; IHD, ischemic heart disease; CHF, congestive heart failure; COPD, chronic obstructive pulmonary disease; LC, liver cirrhosis.

### Subgroup analyses

**Figure 4:**
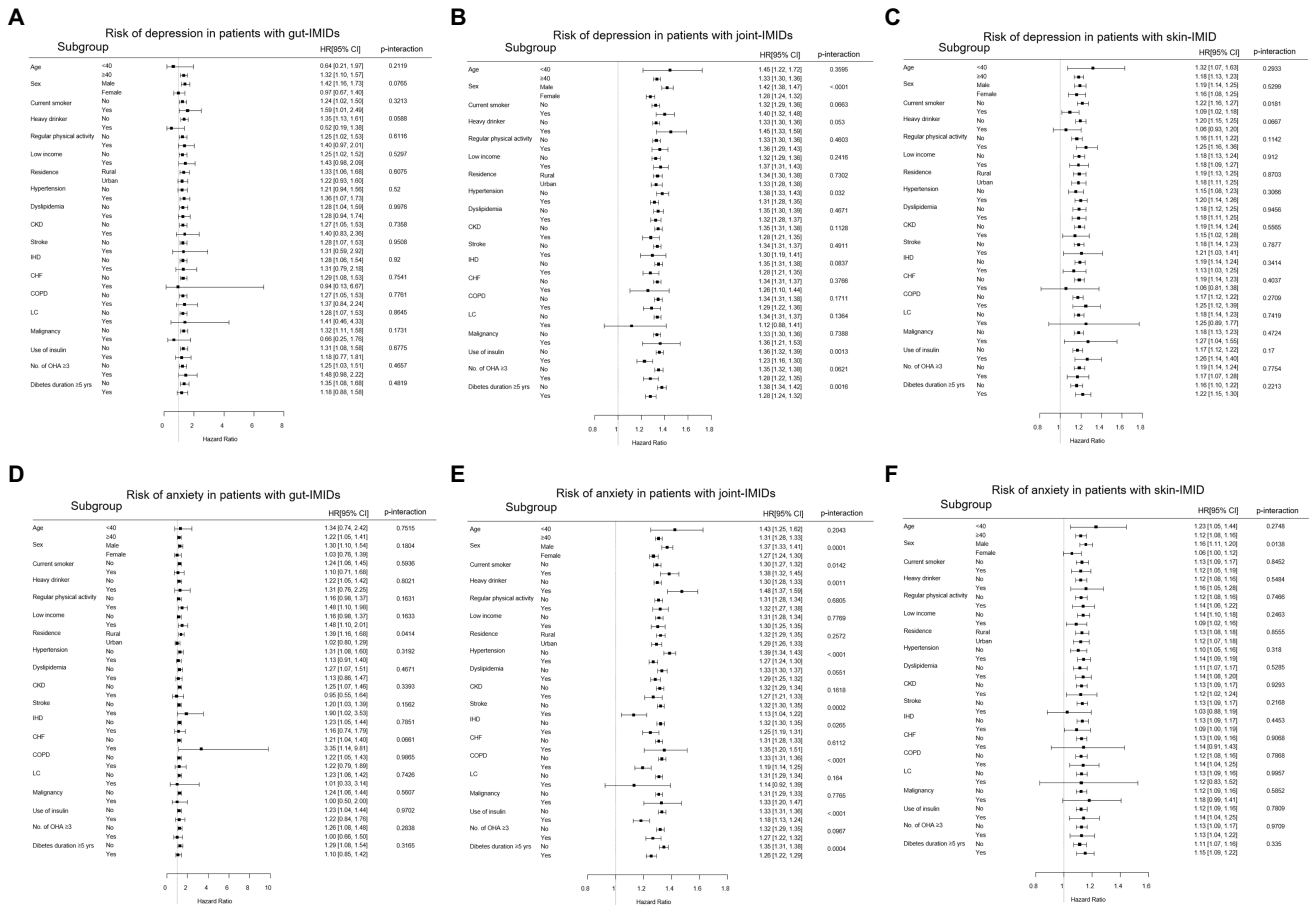
Subgroup analyses for the risk of depression in patients with **(A)** gut IMIDs, **(B)** joint IMIDs, and **(C)** skin IMID; and the risk of anxiety in patients with **(D)** gut IMIDs, **(E)** joint IMIDs, and **(F)** skin IMID. IMIDs, immune-mediated inflammatory disease; CKD, chronic kidney disease; IHD, ischemic heart disease; CHF, congestive heart failure; COPD, chronic obstructive pulmonary disease; LC, liver cirrhosis; OHA, oral hypoglycemic agents.

The results of the subgroup analyses are shown in Figure 4. The impact of gut IMIDs on the incidence of depression did not differ significantly between subgroups of patients stratified by multiple risk factors (Figure 4A). The effect of joint IMIDs on the incidence of depression was more pronounced in men (*p*-interaction < 0.0001), patients without hypertension (*p*-interaction = 0.032), those not using insulin (*p*-interaction = 0.0013), and those who had a disease duration of T2DM of < 5 years (*p*-interaction = 0.0016; Figure 4B). The influence of skin IMID on the incidence of depression was more prominent in current smokers (*p*-interaction = 0.0181; Figure 4C).

The impact of gut IMIDs on the development of anxiety was more prominent in patients of rural residence (*p*-interaction = 0.0414; Figure 4D). The impact of joint IMIDs on the incidence of anxiety was more prominent in male patients (*p*-interaction = 0.0001), those who were current smokers (*p*-interaction = 0.0142) and heavy alcohol drinkers (*p*-interaction = 0.0011), those without hypertension (*p*-interaction < 0.0001), stroke (p-interaction = 0.0002), ischemic heart disease (*p*-interaction = 0.0265) and COPD (*p*-interaction < 0.0001), patients not using insulin (*p*-interaction < 0.0001), and those who had a disease duration of T2DM of <5 years (*p*-interaction = 0.0004; Figure 4E). The influence of skin IMID on the incidence of anxiety was more prominent in men (*p*-interaction = 0.0138; Figure 4F).

**Figure 5:**
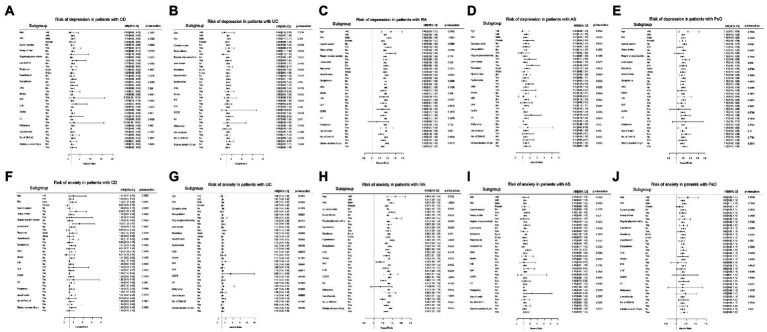
Subgroup analyses for the risk of depression in patients with **(A)** CD, **(B)** UC, **(C)** RA, **(D)** AS, and **(E)** PsO; and the risk of anxiety in patients with **(F)** CD, **(G)** UC, **(H)** RA, **(I)** AS, and **(J)** PsO. CD, crohn’s disease; UC, ulcerative colitis; RA, rheumatoid arthritis; AS, ankylosing spondylitis; PsO, psoriasis; CKD, chronic kidney disease; IHD, ischemic heart disease; CHF, congestive heart failure; COPD, chronic obstructive pulmonary disease; LC, liver cirrhosis; OHA, oral hypoglycemic agents.

The subgroup analysis for the influence of individual IMIDs on the development of depression and anxiety is shown in Figure 5.

## Discussion

In this large-scale population-based study, we found that existence of gut IMIDs, joint IMIDs, and skin IMID is associated with a significantly higher risk of developing depression and anxiety in patients with T2DM as compared to their respective counterparts that did not have those IMIDs. Moreover, according to the number of IMIDs present, patients with T2DM who had ≥ 2 IMIDs had a higher risk of depression and anxiety than those who had one IMID, both of whom had higher risk than those without any IMIDs. We also identified subgroups of patients in whom the impacts of IMIDs were more prominent. Our findings are noteworthy because it is the first to comprehensively assess the effect of organ-based IMIDs on depression and anxiety in patients with T2DM, which are associated with poor outcomes in patients with T2DM (42–44). These data provide better insights in stratifying the risk of depression and anxiety in patients with T2DM, which can lead to closer monitoring for depression and anxiety in high risk patients.

According to the existence of IMIDs in an organ-based manner, joint IMIDs had the largest effect sizes on depression and anxiety (adjusted HRs 1.335 and 1.311, respectively), followed by the gut IMIDs (adjusted HRs of 1.284 and 1.223, respectively). Skin IMID had the smallest effect sizes on depression and anxiety (adjusted HRs of 1.183 and 1.126, respectively). Considering that patients with T2DM are already at a higher risk of depression (1.67-fold higher risk) and anxiety (1.73-fold higher risk) compared with the general population (1, 2), the HRs of each organ-based IMID are substantial. These associations reflect that comorbid chronic inflammatory disorders are crucial factors associated with psychological distress in patients with T2DM, while the additional risks may be different depending on the affected organs.

Similar with the analyses where depression and anxiety were analyzed separately, when association between IMIDs and co-occurrence of depression and anxiety was assessed, joint IMIDs and skin IMID were significantly associated with a higher risk co-occurrence of depression and anxiety, and gut IMIDs had a trend of being associated with a higher risk co-occurrence of depression and anxiety. Given that IMIDs are chronic diseases (4), these results are accordant with the previous knowledge that depression and anxiety commonly co-occur in patients with chronic disease, such as COPD (45). Then, a question rises on whether depression and anxiety affect one another. When incident anxiety was additionally adjusted in the analyses of incident depression (model 7 in Table 2 and Table 3), and vice versa (model 7 in Table 4 and Table 5), although the effect sizes were attenuated, similar associations were observed as in model 6, suggesting that the associations of IMIDs with incident depression or anxiety are independent of incident anxiety or depression, respectively.

Mechanistically, a link between inflammatory process and neuropsychiatric conditions have been consistently reported (46, 47). In inflammatory conditions, pro-inflammatory cytokines access the central nervous system and interact with a cytokine network in the brain, influencing the brain function relevant to the development of psychological distress including depression and anxiety (48). Moreover, from an organ-based perspective, joint-brain axis, gut-brain axis, and gut-brain-skin axis have been proposed to explain the crosstalk of joint, gut, and skin inflammation with psychological distress, respectively (49–51). Together with this background knowledge, our data suggest that it is important to perceive IMIDs, a disease group typified by chronic inflammation (4), as risk factors of depression and anxiety in patients with T2DM.

The HRs for depression and anxiety were higher in patients with T2DM who had ≥ 2 IMIDs (42% higher risk and 49% higher risk, respectively) than in those with one IMID (30% higher risk and 26% higher risk, respectively). This suggests that higher inflammatory burden could be associated with a higher risk of developing depression and anxiety. Indeed, previous studies have shown that concentrations of inflammatory markers such as C-reactive protein and interleukin-6 correlate with the scores of depression and anxiety, supporting our findings (52, 53). Based on our data, the risk of depression and anxiety in patients with T2DM can be stratified in the following order: patients with ≥ 2 IMIDs, those with joint IMIDs, those with gut IMIDs, and those with skin IMID.

When individual IMIDs were analyzed separately, Crohn’s disease had a trend of being associated with a higher risk of depression and anxiety, but did not reach statistical significance. The wide 95% CIs of the HRs of Crohn’s disease suggest that this is probably due to the small number of patients with CD. The HR itself (48% higher risk and 33% higher risk for depression and anxiety, respectively), was numerically the highest compared to that of every other IMID. Therefore, although the association between Crohn’s disease and risk of depression and anxiety did not reach statistical significance, patients with Crohn’s disease could still be considered as having higher risk of depression and anxiety clinically.

Interestingly, ankylosing spondylitis showed a trend of being associated with a lower risk of anxiety in the univariable model, which was not observed in other individual IMIDs. We assume that this finding is attributable to the high proportion of males in patients with ankylosing spondylitis. The proportion of males (91.7%) was numerically the highest in patients with ankylosing spondylitis, compared with patients with other IMIDs. Given that female sex is a risk factor of anxiety (54, 55), the high proportion of males (i.e., low proportion of females) in patients with ankylosing spondylitis could explain why these patients showed a trend for a lower risk of anxiety. Indeed, this association was reversed in the subsequent models (model 2–6) where sex was adjusted and showed a trend of being associated with a higher risk of anxiety.

Some limitations should be noted in our study. First, this was a retrospective observational study. Although we found interesting associations between IMIDs and depression and anxiety, causality cannot be inferred. In addition, we lack mechanistic explanations for why some IMIDs pose higher risk than the others and why the impacts of IMIDs are more pronounced in particular subgroups of patients. Second, as this was a Korean nationwide population-based study, it is unclear whether our results are generalizable to different ethnic populations. Third, we lack data on the severity of IMIDs and were unable to analyze the association between the severity of IMIDs and depression and anxiety. Fourth, markers of inflammation such as erythrocyte sedimentation rate and C-reactive protein were unavailable in the NHIS database. Fifth, we could not determine whether comorbid IMIDs lower the threshold for psychological distress screening in this study, because the primary outcome was defined using ICD-10 codes from the claims database. Sixth, the diagnoses of depression and anxiety were not systematically screened and detected. Under-diagnosis of mental disorders is common in primary care and therefore, there is a possibility of bias resulting from under-diagnosis of depression and anxiety. Despite these limitations, the use of a nationwide database that covers the entire Korean population allowed for a large sample of patients with T2DM and an adequate number of outcome events, which are usually difficult to achieve in clinical trials. This enabled us to sufficiently evaluate the impact of IMIDs on the risk of depression and anxiety in patients with T2DM.

**Figure 6:**
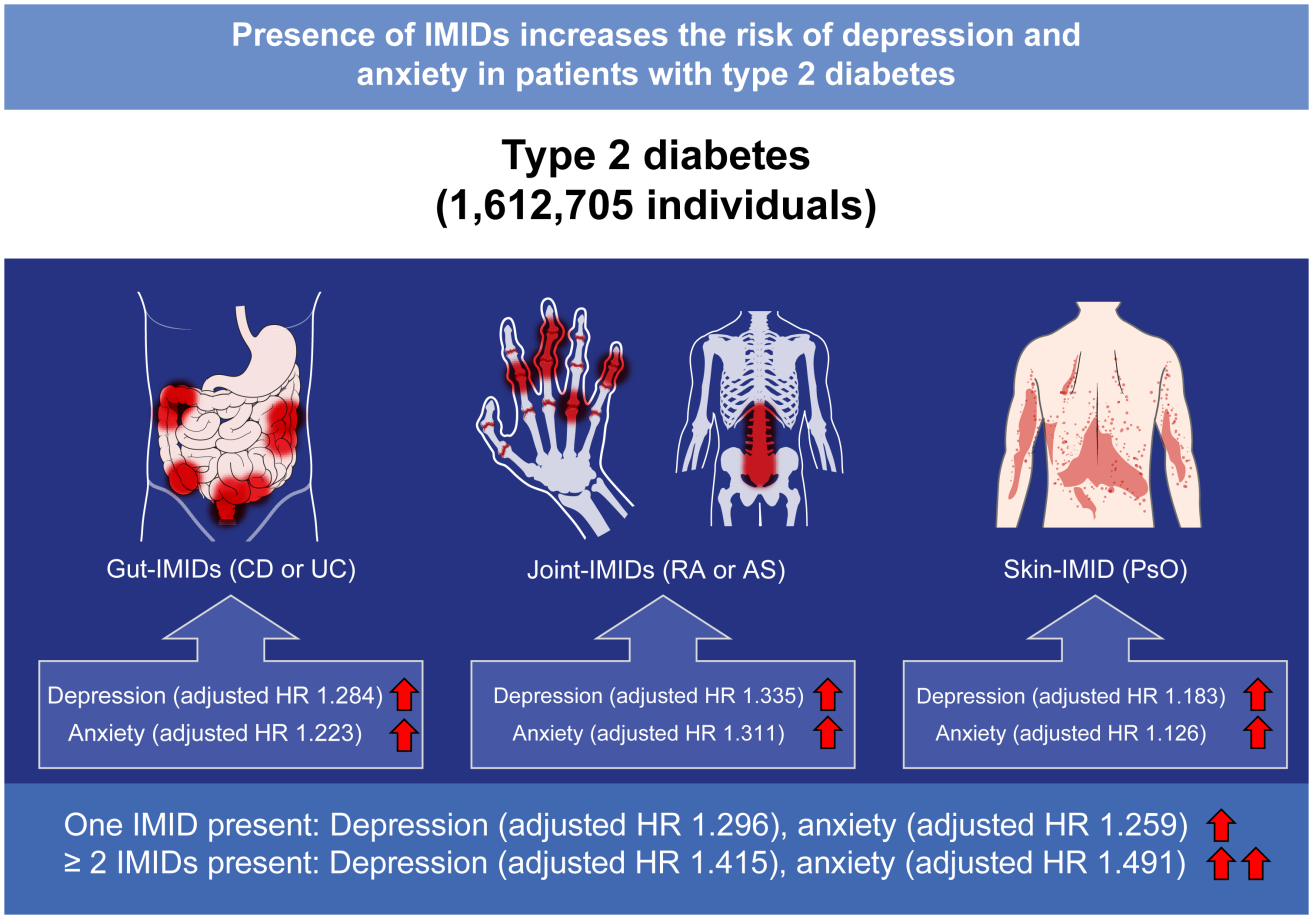
Summary of the present study. IMIDs, immune-mediated inflammatory disease; CD, Crohn’s disease; UC, ulcerative colitis; RA, rheumatoid arthritis; AS, ankylosing spondylitis; PsO, psoriasis; HR, hazard ratio.

In conclusion, in patients with T2DM, the presence of IMIDs was associated with higher risks of depression and anxiety (Figure 6). The risk was higher in patients with ≥ 2 IMIDs than in those with one IMID. According to organ-based classification, joint IMIDs posed the highest risk, followed by gut IMIDs, and then the skin IMID. Our data suggest that patients with T2DM with concomitant IMIDs should be recognized as high risk patients for developing depression and anxiety, thus needing more stringent screening for depression and anxiety. This is of importance given the clinical implication that psychological distress has on patient-reported outcomes and prognosis of T2DM. Further prospective studies evaluating optimal screening schedule for depression and anxiety in these high risk patients are warranted.

## Data availability statement

The original contributions presented in the study are included in the article/Supplementary material, further inquiries can be directed to the corresponding authors.

## Ethics statement

The studies involving human participants were reviewed and approved by Institutional Review Board (IRB) of Gangnam Severance Hospital (IRB No: 3-2020-0269). Written informed consent for participation was not required for this study in accordance with the national legislation and the institutional requirements.

## Author contributions

OK, YK, JC, and KH: concept, design, administrative, and technical or material support. OK, YK, JC, KH, M-CP, and RK: acquisition, analysis, or interpretation of data. OK, YK, JC, and KH: drafting of the manuscript. OK, YK, JC, KH, M-CP, RK, J-HK, YY, and HP: critical revision of the manuscript for important intellectual content. KH: statistical analysis. OK: obtained funding. JC and KH: supervision. All authors contributed to the article and approved the submitted version.

## Funding

This work was supported by the research fund of Rheumatology Research Foundation (RRF-2022-02).

## Conflict of interest

The authors declare that the research was conducted in the absence of any commercial or financial relationships that could be construed as a potential conflict of interest.

## Publisher’s note

All claims expressed in this article are solely those of the authors and do not necessarily represent those of their affiliated organizations, or those of the publisher, the editors and the reviewers. Any product that may be evaluated in this article, or claim that may be made by its manufacturer, is not guaranteed or endorsed by the publisher.
