# Supplementary material for: Association of immune-mediated inflammatory diseases with depression and anxiety in patients with type 2 diabetes: A nationwide population-based study
Source: Front Med (Lausanne). 2023 Apr 17;10:1103911. doi: 10.3389/fmed.2023.1103911 (PMC10150640; doi:10.3389/fmed.2023.1103911)
Supplement: Supplementary file 1 [file Table_1.DOCX]

**Article Title: Association of immune-mediated inflammatory diseases with depression and anxiety in patients with type 2 diabetes: A nationwide population-based study**

Supplemental Table 1. Incidence rates of depression stratified by specific diagnoses

| Depression | N | F32 | Duration | IR (per 1,000) | F33 | Duration | IR (per 1,000) |
| --- | --- | --- | --- | --- | --- | --- | --- |
| Gut IMIDs |  |  |  |  |  |  |  |
| No | 1612067 | 248066 | 10322330.67 | 24.032 | 9952 | 10322330.67 | 0.96412 |
| Yes | 638 | 120 | 3990.72 | 30.0698 | 5 | 3990.72 | 1.25291 |
| Joint IMIDs |  |  |  |  |  |  |  |
| No | 1581324 | 240531 | 10139725.04 | 23.7216 | 9593 | 10139725.04 | 0.94608 |
| Yes | 31381 | 7655 | 186596.34 | 41.0244 | 364 | 186596.34 | 1.95073 |
| Skin IMID |  |  |  |  |  |  |  |
| No | 1599605 | 245733 | 10244787.62 | 23.9861 | 9864 | 10244787.62 | 0.96283 |
| Yes | 13100 | 2453 | 81533.77 | 30.0857 | 93 | 81533.77 | 1.14063 |
| Number of IMIDs |  |  |  |  |  |  |  |
| 0 | 1567988 | 238061 | 10056564.14 | 23.6722 | 9499 | 10056564.14 | 0.94456 |
| 1 | 44206 | 10005 | 266715.84 | 37.5118 | 454 | 266715.84 | 1.70219 |
| ≥2 | 511 | 120 | 3041.41 | 39.4554 | 4 | 3041.41 | 1.31518 |
| Crohn’s disease |  |  |  |  |  |  |  |
| No | 1612612 | 248169 | 10325763.77 | 24.034 | 9957 | 10325763.77 | 0.96429 |
| Yes | 93 | 17 | 557.62 | 30.4868 | 0 | 557.62 | 0 |
| Ulcerative colitis |  |  |  |  |  |  |  |
| No | 1612156 | 248081 | 10322869.77 | 24.0322 | 9952 | 10322869.77 | 0.96407 |
| Yes | 549 | 105 | 3451.62 | 30.4205 | 5 | 3451.62 | 1.4486 |
| Rheumatoid arthritis |  |  |  |  |  |  |  |
| No | 1581729 | 240596 | 10142313.25 | 23.722 | 9597 | 10142313.25 | 0.94623 |
| Yes | 30976 | 7590 | 184008.13 | 41.2482 | 360 | 184008.13 | 1.95644 |
| Ankylosing spondylitis |  |  |  |  |  |  |  |
| No | 1612187 | 248105 | 10323036.39 | 24.0341 | 9953 | 10323036.39 | 0.96415 |
| Yes | 518 | 81 | 3285 | 24.6576 | 4 | 3285 | 1.21766 |
| Psoriasis |  |  |  |  |  |  |  |
| No | 1599605 | 245733 | 10244787.62 | 23.9861 | 9864 | 10244787.62 | 0.96283 |
| Yes | 13100 | 2453 | 81533.77 | 30.0857 | 93 | 81533.77 | 1.14063 |

IR, incidence rate; IMIDs, immune-mediated inflammatory diseases

Supplemental Table 2. Incidence rates of anxiety stratified by specific diagnoses

| Anxiety | N | F40 | Duration | IR (per 1,000) | F41 | Duration | IR (per 1,000) |
| --- | --- | --- | --- | --- | --- | --- | --- |
| Gut IMIDs |  |  |  |  |  |  |  |
| No | 1612067 | 6683 | 9714870.15 | 0.6879 | 388486 | 9714870.15 | 39.9888 |
| Yes | 638 | 5 | 3742.99 | 1.3358 | 175 | 3742.99 | 46.7541 |
| Joint IMIDs |  |  |  |  |  |  |  |
| No | 1581324 | 6499 | 9548922.79 | 0.6806 | 377342 | 9548922.79 | 39.5167 |
| Yes | 31381 | 189 | 169690.34 | 1.1138 | 11319 | 169690.34 | 66.7039 |
| Skin IMID |  |  |  |  |  |  |  |
| No | 1599605 | 6624 | 9642017.01 | 0.687 | 385086 | 9642017.01 | 39.9383 |
| Yes | 13100 | 64 | 76596.13 | 0.8356 | 3575 | 76596.13 | 46.6734 |
| Number of IMIDs |  |  |  |  |  |  |  |
| 0 | 1567988 | 6431 | 9470662.27 | 0.679 | 373753 | 9470662.27 | 39.4643 |
| 1 | 44206 | 256 | 245212.66 | 1.044 | 14724 | 245212.66 | 60.0458 |
| ≥2 | 511 | 1 | 2738.21 | 0.3652 | 184 | 2738.21 | 67.1972 |
| Crohn’s disease |  |  |  |  |  |  |  |
| No | 1612612 | 6687 | 9718082.02 | 0.6881 | 388637 | 9718082.02 | 39.9911 |
| Yes | 93 | 1 | 531.12 | 1.8828 | 24 | 531.12 | 45.1875 |
| Ulcerative colitis |  |  |  |  |  |  |  |
| No | 1612156 | 6684 | 9715380.63 | 0.688 | 388509 | 9715380.63 | 39.9891 |
| Yes | 549 | 4 | 3232.51 | 1.2374 | 152 | 3232.51 | 47.0223 |
| Rheumatoid arthritis |  |  |  |  |  |  |  |
| No | 1581729 | 6501 | 9551378.85 | 0.6806 | 377432 | 9551378.85 | 39.516 |
| Yes | 30976 | 187 | 167234.29 | 1.1182 | 11229 | 167234.29 | 67.1453 |
| Ankylosing spondylitis |  |  |  |  |  |  |  |
| No | 1612187 | 6686 | 9715477.15 | 0.6882 | 388548 | 9715477.15 | 39.9927 |
| Yes | 518 | 2 | 3135.99 | 0.6378 | 113 | 3135.99 | 36.0333 |
| Psoriasis |  |  |  |  |  |  |  |
| No | 1599605 | 6624 | 9642017.01 | 0.687 | 385086 | 9642017.01 | 39.9383 |
| Yes | 13100 | 64 | 76596.13 | 0.8356 | 3575 | 76596.13 | 46.6734 |

IR, incidence rate; IMIDs, immune-mediated inflammatory diseases
